# Association of elevated serum active IL-18 levels with cytokine profiles and clinical features in adult-onset Still’s disease

**DOI:** 10.3389/fimmu.2026.1759584

**Published:** 2026-04-28

**Authors:** Shuhei Yoshida, Haruki Matsumoto, Yuya Fujita, Yuya Sumichika, Kenji Saito, Tomoyuki Asano, Shuzo Sato, Masashi Mizokami, Masaya Sugiyama, Yasuhiro Shimojima, Atsushi Kawakami, Kiyoshi Migita

**Affiliations:** 1 Department of Rheumatology, Fukushima Medical University School of Medicine, Fukushima, Fukushima, Japan; 2 Department of Clinical Immunology, Graduate School of Medicine, Osaka Metropolitan University, Osaka, Japan; 3 Genome Medical Sciences Project, National Institute of Global Health and Medicine, Japan Institute for Health Security, Ichikawa, Chiba, Japan; 4 Department of Viral Pathogenesis and Controls, National Institute of Global Health and Medicine, Japan Institute for Health Security, Ichikawa, Chiba, Japan; 5 Department of Immunology and Rheumatology, Division of Advanced Preventive Medical Sciences, Nagasaki University Graduate School of Biomedical Sciences, Nagasaki, Japan; 6 Innovation Platform & Office for Precision Medicine, Nagasaki University Graduate School of Biomedical Sciences, Nagasaki, Japan; 7 Clinical Research Center, Nagasaki Medical Center, Omura, Nagasaki, Japan; 8 Department of Rheumatology, St. Francis Hospital, Nagasaki, Nagasaki, Japan

**Keywords:** adult-onset still’s disease, autoinflammatory disease, cytokine profiles, IL-18, inflammasome, macrophage activation syndrome

## Abstract

**Introduction:**

Inflammasome-mediated activation of interleukin (IL)-1β and IL-18 plays a key role in the pathogenesis of adult-onset Still’s disease (AOSD), a systemic autoinflammatory disorder. The cleaved free active form of IL-18 may more accurately reflect inflammasome activity than total IL-18, which includes pro–IL-18, free active IL-18, and IL-18 bound to IL-18–binding protein. This study aimed to measure serum active IL-18 levels in patients with AOSD and evaluate their clinical and diagnostic significance.

**Methods:**

Serum samples were obtained from 47 untreated patients with AOSD, 42 patients with rheumatoid arthritis (RA), 9 patients with familial Mediterranean fever (FMF), and 26 healthy controls (HCs). Active IL-18 was quantified using a specific ELISA detecting cleaved, biologically active IL-18, and 69 cytokines were analyzed in patients with AOSD using a multiplex suspension array. Correlations between active IL-18 and other cytokines, clinical parameters, and the reactive hemophagocytic syndrome diagnostic score (HScore) were evaluated, and discriminative performance was assessed using receiver operating characteristic analysis.

**Results:**

Serum levels of active IL-18 were significantly higher in patients with AOSD than in those with RA, FMF, and HCs (all p < 0.001). Active IL-18 levels positively correlated with the Pouchot score (p < 0.001), serum ferritin level (p = 0.004), and C-reactive protein level (p = 0.004) and decreased significantly after immunosuppressive therapy (p = 0.003). Active IL-18 significantly and positively correlated with total IL-18, macrophage colony-stimulating factor, basic fibroblast growth factor, leukemia inhibitory factor, chemokine (C-X-C motif) ligand 9, and IL-12 (p40), all of which were significantly correlated with the HScore. Elevated active IL-18 levels were associated with rashes and splenomegaly. Among the evaluated biomarkers, active IL-18 showed the highest diagnostic accuracy for AOSD (cutoff > 4,231.1 pg/mL; sensitivity, 89.4%; specificity, 92.9%; area under the curve 0.963).

**Conclusions:**

Serum active IL-18 levels were significantly higher in patients with AOSD than in HCs and in patients with FMF or RA. Active IL-18 levels were associated with clinical features such as rash and splenomegaly and correlated with specific cytokines. Serum active IL-18 levels may serve as a biomarker of inflammasome activation and disease activity in patients with AOSD.

## 1 Introduction

Adult-onset Still’s disease (AOSD) is a systemic autoinflammatory disease accompanied by skin rashes, spiking fever, arthritis, sore throat, lymphadenopathy and macrophage activation syndrome (MAS) (1, 2). These various organ involvements represent heterogeneous clinical phenotypes, and early diagnosis and discrimination of the disease phenotype remain critical for improving the long-term outcomes of AOSD (3, 4). Serum biomarkers are useful tools that offer significant advantages in determining disease activity or phenotype and therapeutic decisions, such as cytokine-targeting therapy, in the management of AOSD (5). Several inflammatory molecules have been proposed as diagnostic biomarkers for AOSD. Among these, serum IL-18 is the most studied biomarker of AOSD (6).

Although the pathogenesis of AOSD remains to be completely elucidated, inflammasome activation is considered a hallmark of AOSD (5). Nucleotide-binding oligomerization domain, leucine-rich repeat, and pyrin domain 3 (NLRP3) inflammasome activation triggers caspase-1 activation, converting pro-interleukin (IL)-1β and pro-IL-18 into their active forms (7), which are key cytokines involved in the pathogenesis of AOSD (1, 8). The active form of IL-18 seems to be a valuable biomarker for monitoring the inflammasome activation status; therefore, direct detection of the active form of IL-18 is critical in inflammasome-mediated autoinflammation in AOSD.

Urano et al. generated monoclonal antibodies that allowed the detection of the cleaved, active form of IL-18 (not the inactive precursor IL-18) and established an enzyme-linked immunosorbent assay (ELISA) detection system for active forms of IL-18 (9). This study aimed to evaluate the serum levels of active IL-18 in patients with active AOSD and assess the diagnostic value of serum active IL-18 level as an inflammatory biomarker of AOSD.

## 2 Materials and methods

### 2.1 Patients and study design

We previously identified cytokines and chemokines that distinguish the clinical phenotype of AOSD from other conditions in patients who visited our institution (10). In this study, residual serum samples previously collected from patients with AOSD were used. Of the selected 49 patients, samples from two were unavailable, resulting in the analysis of 47 patients. A total of 47 untreated patients with AOSD who visited the Department of Rheumatology, Fukushima Medical University Hospital, between January 1995 and August 2022 were enrolled in this study. Eligible patients were aged ≥18 years and met the diagnostic criteria for AOSD established by Yamaguchi et al. (11), after excluding those with infectious, neoplastic, or other autoimmune diseases. Pre-treatment serum samples were collected from 47 patients with AOSD who were enrolled in the study. In addition, serum samples were obtained from 18 treated patients in remission to evaluate longitudinal changes. The timing of serum sample collection and the treatments administered at the time of sampling in the 18 patients with AOSD after treatment initiation are shown in **Supplementary Table 1**. Disease activity was assessed using the Pouchot score (12). MAS was diagnosed according to the European League Against Rheumatism (EULAR)/American College of Rheumatology (ACR)/Pediatric Rheumatology International Trials Organization (PRINTO) classification criteria (13) and the reactive hemophagocytic syndrome diagnostic score (HScore) proposed by Fardet et al. (14).

To evaluate the disease specificity of active IL-18 in AOSD, patients with rheumatoid arthritis (RA) and familial Mediterranean fever (FMF) were included as controls. An additional independent dataset comprised 42 patients with RA and 9 patients with FMF, none of whom had inflammatory comorbidities such as malignancy, infection, or interstitial lung disease. Among patients diagnosed with RA at Fukushima Medical University Hospital, those who were asymptomatic or in complete remission were excluded, and patients with clinically active disease were randomly selected. All patients met the 2010 ACR/EULAR classification criteria for RA (15). Serum samples were collected from patients with RA, regardless of treatment status. FMF patients were diagnosed at Fukushima Medical University Hospital according to the Tel-Hashomer diagnostic criteria (16). For FMF patients, serum samples were collected during disease attacks, irrespective of treatment status. Healthy controls (HCs) were recruited from the staff of the Department of Rheumatology, Fukushima Medical University, between April 2021 and April 2023. The control group included 10 men and 16 women aged 20–60 years, and none had an active illness or were receiving medical treatment.

An opt-out strategy was selected for the participants, and those who declined to provide informed consent were excluded. This study was approved by the Institutional Review Board of Fukushima Medical University (No. 2021-290) and the National Center for Global Health and Medicine (NCGM-187).

### 2.2 Chemokine and cytokine measurements

The experimental procedure was performed as previously described (10). The Bio-Plex 3D system (Bio-Rad, Hercules, CA, USA) was used to perform multiplex assays of humoral factors according to the manufacturer’s instructions. Briefly, the serum samples were analyzed using the Bio-Plex 3D system and a Bio-Plex Pro Wash Station equipped with a magnetic manifold (17). Cytokine concentrations were calculated from the standard curves generated for each assay plate and expressed as serum cytokine or chemokine levels (pg/mL). The analyses included the use of the Bio-Plex Pro human cytokine screening 48-Plex and Bio-Plex Pro human chemokine screening 40-Plex. Additionally, C-C motif ligand (CCL)17 and interferon (IFN)-λ3 levels were measured using the HISCL-5000 analyzer (Sysmex Corporation, Kobe, Japan) (17).

Furthermore, the following 69 humoral factors were measured using this assay: IL-1α, IL-1β, IL-1Ra, IL-2, IL-2Ra, IL-3, IL-4, IL-5, IL-6, IL-7, IL-8/CXCL8, IL-9, IL-10, IL-12(p40), IL-12(p70), IL-13, IL-15, IL-16, IL-17, total IL-18, 6Ckine/chemokine CCL21, B cell-attracting chemokine-1/chemokine (C-X-C motif) ligand (CXCL) 13, cutaneous T cell-attracting chemokine CCL27, epithelial-derived neutrophil-activating protein-78/CXCL5, eotaxin/CCL11, eotaxin-2/CCL24, eotaxin-3/CCL26, fractalkine/CX3CR1-ligand, granulocyte chemotactic protein-2/CXCL6, granulocyte colony-stimulating factor (CSF), granulocyte macrophage CSF, macrophage CSF (M-CSF), growth-regulated protein-α/CXCL1, growth-regulated protein-β/CXCL2, IFN-α2, IFN-γ, IFN-λ3, I-309/CCL1, IFN-inducible T cell alpha chemoattractant/CXCL11, interferon γ-induced protein-10/CXCL10, monocyte chemotactic protein (MCP)-1/CCL2, MCP-2/CCL8, MCP-3/CCL7, MCP-4/CCL13, macrophage-derived chemokine/CCL22, macrophage migration inhibitory factor, monokine induced by interferon-γ/CXCL9, macrophage inflammatory protein (MIP)-1α/CCL3, MIP-1δ/CCL15, MIP-3α/CCL20, MIP-3β/CCL19, myeloid progenitor inhibitor factor-1/CCL23, small-inducible cytokine B16/CXCL16, stromal derived factor-1α+β/CXCL12, thymus-expressed chemokine/CCL25, tumor necrosis factor (TNF)-α, TNF-β, basic fibroblast growth factor (FGF), hepatocyte growth factor, leukemia inhibitory factor (LIF), MIP-1β, platelet derived growth factor-BB, regulated on activation, normal T cell expressed and secreted, stem cell factor, stem cell growth factor-β, TNF-related apoptosis-inducing ligand, vascular endothelial growth factor, β-nerve growth factor, and thymus and activation-regulated chemokine/CCL17. In this study, total IL-18 was defined as the sum of pro–IL-18, active IL-18, and IL-18 bound to IL-18–binding protein (IL-18BP).

### 2.3 Measurement of serum concentration of active IL-18

Serum levels of active IL-18 were assessed using a commercially available ELISA kit (Catalog No. E-I-002, Human Activated IL-18 ELISA Assay Kit Ver.2, mAbProtein Co. Ltd., Izumo, Shimane, Japan), which can detect active IL-18. This ELISA kit employs monoclonal antibody 9-10.2, which specifically recognizes human active IL-18 as the capture antibody and enzyme-labeled anti-human IL-18 rabbit polyclonal antibody as the detection antibody and uses recombinant human active IL-18^37–193^ expressed and purified from *Escherichia coli* as the assay standard (9, 18). The binding epitope of monoclonal antibody 9-10.2 on IL-18 overlaps with the interaction interface between IL-18 and IL-18BP. Therefore, antibody 9-10.2 has been shown to cannot bind to the IL-18/IL-18BP complex (9). Active IL-18 concentrations were calculated using standard curves following the manufacturer’s instructions. Using this ELISA kit, the lower limit of detection for active IL-18 was 50 pg/mL.

### 2.4 Statistical analysis

Continuous variables were expressed as medians with interquartile ranges (IQRs) and categorical variables as frequencies and percentages. Group differences among patients with AOSD, RA, and HCs were analyzed using the Mann–Whitney U test. The pre- and post-treatment differences were evaluated using the Wilcoxon signed-rank test. Correlations between serum markers were assessed using Spearman’s rank correlation test. A logistic regression model was used to identify cytokine markers and clinical laboratory parameters with optimal diagnostic performance for AOSD. Sensitivity, specificity, receiver operating characteristic (ROC) curves, and areas under the curve (AUCs) were calculated.

Statistical analyses were performed using R version 4.3.1 (R Foundation for Statistical Computing, Vienna, Austria) and SPSS version 29.0 (IBM Corp., Armonk, NY, USA). All p-values were two-sided, and p < 0.05 was considered statistically significant. The Bonferroni correction was applied for multiple comparisons among the four groups (AOSD, RA, FMF, and HCs) and among the three AOSD subtypes, with statistical significance defined as p < 0.0125 and p < 0.0167, respectively. For multiple cytokine analyses (n = 69), Bonferroni correction was applied, and p < 0.0007 was considered significant.

## 3 Results

### 3.1 Patients, controls, and samples

This study included 47 patients with AOSD, 42 patients with RA and 9 patients with FMF as disease controls, and 26 HCs. **Table 1** summarizes the demographic and clinical characteristics of the patients with AOSD. The median age of the participants was 42 years (IQR 30–60 years) and 32 were female. The clinical subtypes were as follows: polycyclic systemic in 29 patients (61.7%), monocyclic systemic in 14 (29.8%), and chronic articular in four (8.5%). The median Pouchot disease activity score was 3 (IQR 2–5), and MAS was present in seven patients (14.9%). Among patients with AOSD, clinical symptoms and features included fever in 35 patients (74.5%), sore throat in 18 (38.3%), serositis in 8 (17.0%), rash in 31 (66.0%), arthritis in 31 (66.0%), lymphadenopathy in 11 (23.4%), splenomegaly in 18 (38.3%), and hepatomegaly in 18 (38.3%). Relapse during the disease course occurred in 33 patients (70.2%). Serum active IL-18 levels were significantly positively correlated with the Pouchot score, serum ferritin level, and alanine aminotransferase level (p < 0.001, p = 0.001, and p = 0.046, respectively).

**Table 1:** Demographic and clinical characteristics of patients with adult-onset still’s disease at initial diagnosis and their correlations with active IL-18.

Variables	n = 47	Correlation coefficient (95% CI)	p value
Female, n (%)	32 (68.1)		
Age at AOSD diagnosis (years), median (IQR)	42 (30-60)		
Ferritin (ng/mL), median (IQR)	2,722 (426-7,761)	0.46 (0.19-0.66)	0.001^*^
CRP (mg/L), median (IQR)	54.6 (28.5-100.7)	0.24 (-0.06-0.50)	0.1
WBC (/μL), median (IQR)	9,900 (8,000-13,550)	0.09 (-0.21-0.37)	0.56
AST (U/L), median (IQR)	87 (45-187)	0.07 (-0.48-0.57)	0.82
ALT (U/L), median (IQR)	33 (24-88)	0.30 (-0.003-0.55)	0.046^*^
Active IL-18 (pg/mL)	8,592.7 (6,690.3-10,048.6)		
Monocyclic systemic type, n (%)	14 (29.8)		
Polycyclic systemic type, n (%)	29 (61.7)		
Chronic articular type, n (%)	4 (8.5)		
Pouchot score, median (IQR)	3 (2-5)	0.52 (0.27-0.71)	<0.001^*^
Fever, n (%)	35 (74.5)		
Sore throat, n (%)	18 (38.3)		
Serositis, n (%)	8 (17.0)		
Skin rash, n (%)	31 (66.0)		
Arthritis, n (%)	31 (66.0)		
Lymphadenopathy, n (%)	11 (23.4)		
Splenomegaly, n (%)	18 (38.3)		
Hepatomegaly, n (%)	18 (38.3)		
Relapse, n (%)	33 (70.2)		
MAS, n (%)	7 (14.9)		

All data are expressed as median (IQR), or numbers (percentages). * means there is a significant difference at p<0.05.

AOSD, adult-onset Still's disease; ALT, alanine aminotransferase; AST, aspartate aminotransferase; CI, confidence interval; CRP, C-reactive protein; IL, interleukin; IQR, interquartile range; MAS, macrophage activation syndrome; WBC, white blood cell.

The demographic and clinical characteristics of 42 patients with RA and 9 patients with FMF, included as disease controls, as well as 26 HCs are summarized in **Supplementary Table 2**. Among the RA patients, 31 (73.8%) were female, and the median age was 66 years (IQR 58.3–70.0). Most RA patients were receiving disease-modifying antirheumatic drugs, including methotrexate (21/42, 50.0%) and biologic agents (15/42, 35.7%; tocilizumab, n = 5; sarilumab, n = 1; abatacept, n = 6; etanercept, n = 2; and golimumab, n = 1). The median Disease Activity Score in 28 joints using C-reactive protein (DAS28-CRP) was 2.8 (IQR 2.1–4.0). Among patients with FMF during attacks, 4 (44.4%) were female, and the median age was 43 years (IQR 23–52). One patient had typical FMF, whereas 8 had atypical FMF. Five patients were untreated, and 4 received colchicine therapy, including 1 who received combination therapy with colchicine and canakinumab. The baseline demographic and clinical characteristics of the 26 HCs were as follows: 16 (61.5%) were female, and the median age was 32.5 years (IQR 26.3–44.5). Compared with the AOSD group, patients in the RA group were significantly older (p < 0.001), whereas HCs were significantly younger (p = 0.04).

### 3.2 Comparison of active IL-18 levels in patients with AOSD, RA, FMF, and HCs

**Figure 1:**
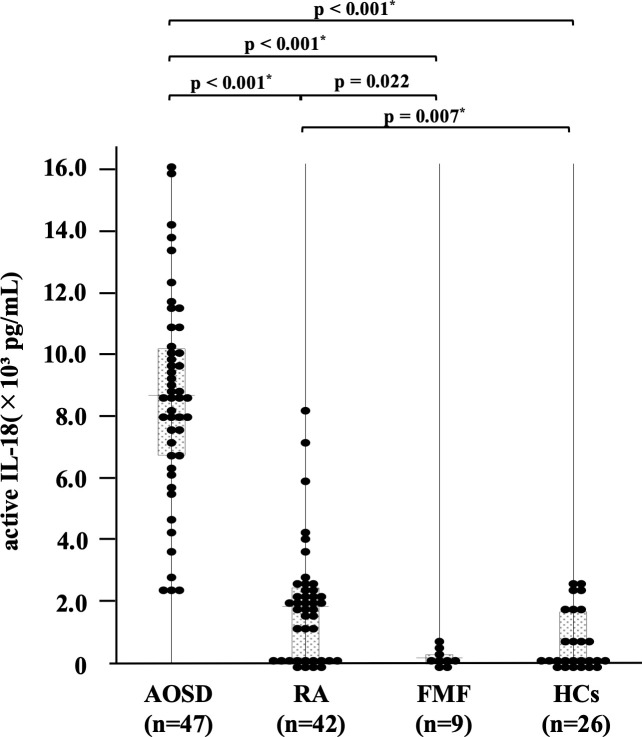
Comparison of serum active IL-18 levels among patients with AOSD, RA, FMF, and HCs. The comparison of serum active IL-18 levels among patients with AOSD (n = 47), RA (n = 42), FMF (n = 9), and HCs (n = 26) is shown. Serum active IL-18 levels were significantly higher in patients with AOSD than in those with RA, FMF, or HCs. AOSD, adult-onset Still’s disease; FMF, familial Mediterranean fever; HC, healthy control; IL, interleukin; RA, rheumatoid arthritis.

We compared the baseline serum levels of active IL-18 (**Figure 1**) between patients with AOSD, RA, FMF, and HCs. Serum active IL-18 levels were significantly higher in AOSD than in RA, FMF, and HCs (all p < 0.001).

**Figure 2:**
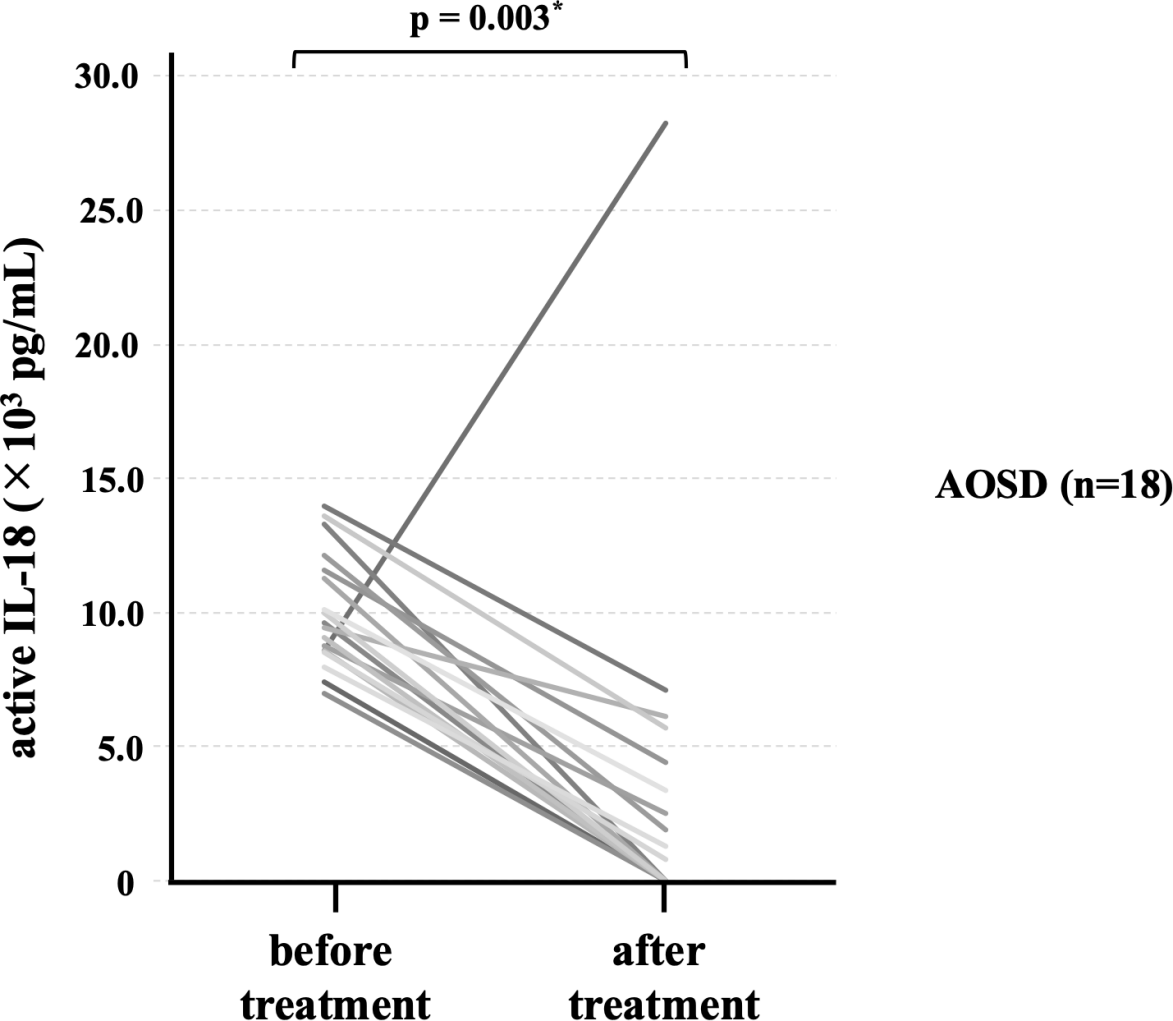
Comparison of serum levels of active IL-18 before and after treatment. Comparison of serum active IL-18 concentrations before and after treatment is shown. Serum active IL-18 levels were significantly lower after treatment than before treatment. IL; interleukin.

To evaluate longitudinal changes in active IL-18 levels, 18 patients with active AOSD were followed until disease activity was controlled, after which repeat blood samples were obtained. The median interval between the initial sampling at AOSD diagnosis and follow-up sampling in these 18 patients was 27 months (IQR 19.8–57 months). Serum active IL-18 levels (**Figure 2**) significantly decreased after immunosuppressive therapy (p = 0.003). After treatment, elevated serum active IL-18 levels were observed in only one female patient with a polycyclic systemic phenotype, in whom follow-up blood sampling was performed 68 months after diagnosis. At the time of sampling, she was 55 years old, with ferritin 384 ng/mL, C-reactive protein 0.9 mg/L, white blood cell count 10,500/μL, aspartate aminotransferase 86 U/L, and alanine aminotransferase 258 U/L, and was receiving prednisolone (20 mg/day) and tacrolimus. She was diagnosed with hepatitis C virus reactivation within one month after follow-up sampling.

### 3.3 Serum levels of active IL-18 in AOSD disease phenotypes, clinical symptoms, and organ lesions

**Figure 3:**
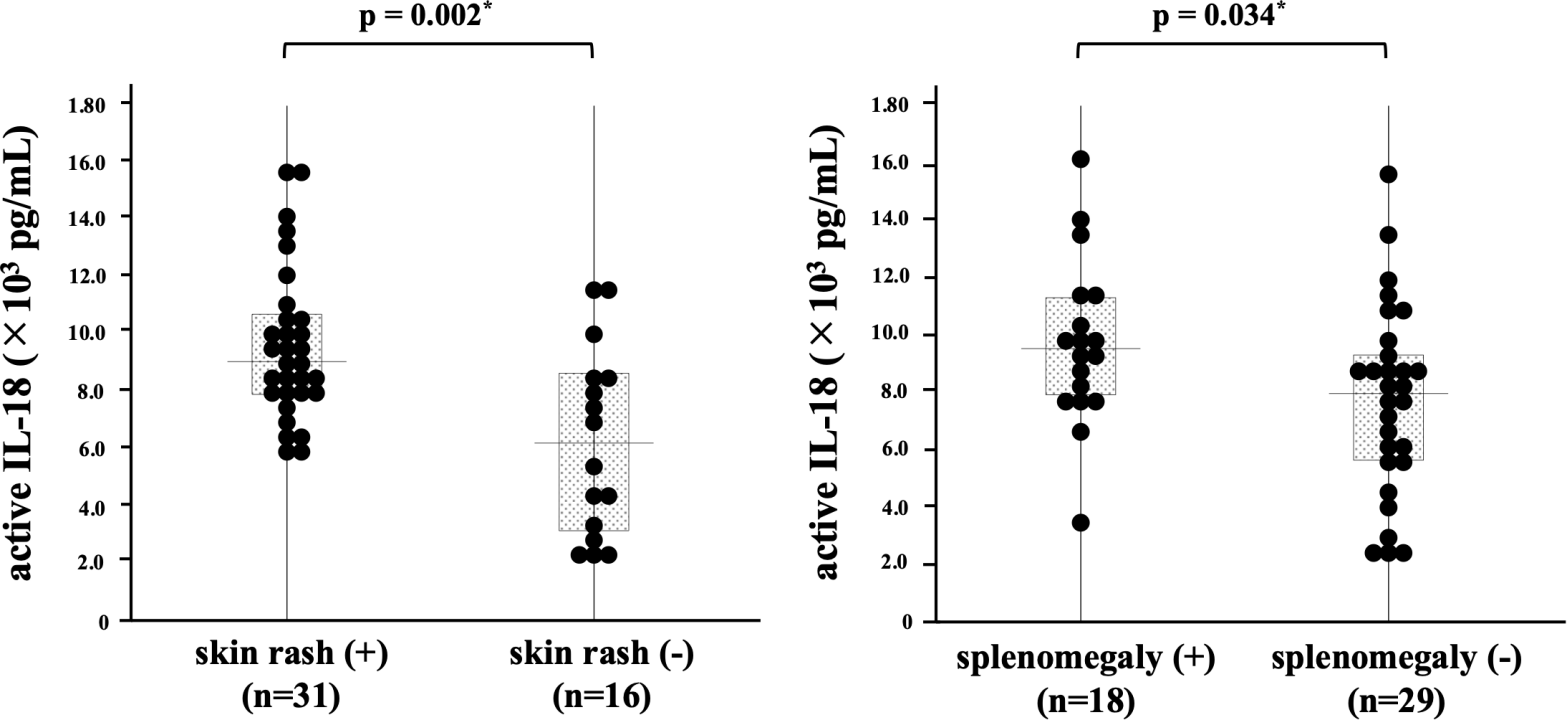
Comparison of serum active IL-18 levels in patients with AOSD with or without skin rash or splenomegaly. Comparisons of serum active IL-18 levels with or without skin rashes or splenomegaly are shown. Serum active IL-18 levels were significantly higher in patients with either clinical feature. AOSD, adult-onset Still’s disease; IL, interleukin.

**Figure 4:**
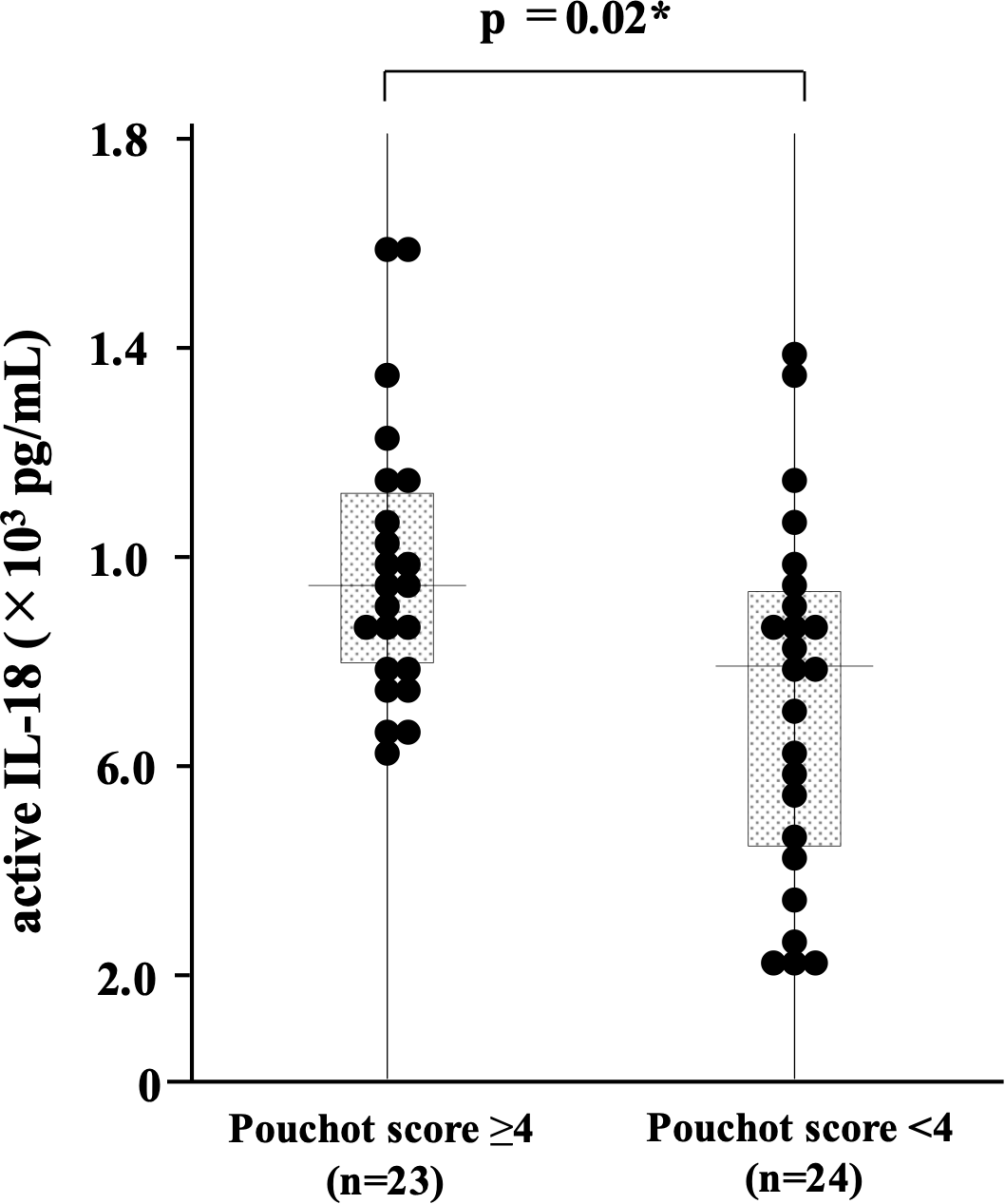
Comparison of serum active IL-18 levels in patients with AOSD according to the Pouchot score. Serum active IL-18 levels were compared between patients with high (≥4) and low (<4) Pouchot scores. Patients with high Pouchot scores (≥4) had significantly higher serum active IL-18 levels. AOSD, adult-onset Still’s disease; IL, interleukin.

We compared the serum levels of active IL-18 between the three AOSD phenotypes (polycyclic systemic, monocyclic systemic, and chronic articular). Additionally, serum levels were analyzed based on the presence or absence of clinical manifestations and organ involvement, including fever, sore throat, serositis, rashes, arthritis, lymphadenopathy, splenomegaly, hepatomegaly, and relapse. In our study, the median Pouchot score among the 47 patients with adult-onset Still’s disease (AOSD) was 3. Active IL-18 levels were compared between patients with a Pouchot score <4 (low Pouchot score group) and those with a Pouchot score ≥4 (high Pouchot score group). No significant differences in serum levels of active IL-18 were observed between the three AOSD phenotypes (data not shown). Similarly, IL-18 levels did not differ significantly in the presence or absence of fever, sore throat, serositis, arthritis, lymphadenopathy, hepatomegaly, or relapse. In contrast, significantly higher levels were observed in patients with rashes and splenomegaly (p = 0.002 and p = 0.034, respectively; **Figure 3**). In addition, active IL-18 levels were significantly elevated in patients in the high Pouchot score group (p = 0.02) (**Figure 4**).

### 3.4 Correlation between active IL-18 and other cytokines in patients with AOSD

We examined the correlation between the serum levels of active IL-18 and other inflammatory cytokines in patients with AOSD. Active IL-18 levels were significantly and positively correlated with total IL-18 (r = 0.62, p < 0.0001), M-CSF (r = 0.62, p < 0.0001), basic FGF (r = 0.58, p < 0.0001), LIF (r = 0.57, p < 0.0001), CXCL9 (r = 0.52, p = 0.0002), and IL-12 (p40) (r = 0.51, p = 0.0003) (**Table 2**). The serum concentrations of these cytokines and chemokines, including active IL-18, are shown in **Supplementary Table 3**.

**Table 2:** The correlations between serum cytokines and active IL-18 in AOSD patients (after Bonferroni`s correction).

Cytokines	Correlation coefficient (95%CI)	p value
Total IL-18	0.62 (0.40-0.78)	<0.0001^*^
M-CSF	0.62 (0.40-0.77)	<0.0001^*^
Basic FGF	0.58 (0.34-0.75)	<0.0001^*^
LIF	0.57 (0.33-0.74)	<0.0001^*^
CXCL9	0.52 (0.26-0.70)	0.0002^*^
IL-12(p40)	0.51 (0.25-0.70)	0.0003^*^

Basic FGF, basic fibroblast growth factor; CI, confidence interval; CXCL, chemokine (C-X-C motif) ligand; IL, interleukin; LIF, leukemia inhibitory factor; M-CSF, macrophage colony-stimulating factor.

* means there is a significant difference at p<0.0007.

### 3.5 Correlations of active IL-18 and six related cytokines with the HScore in patients with AOSD

We examined the correlations between six cytokines that showed significant associations with active IL-18 and the HScore, a predictive score for MAS. Because triglyceride and fibrinogen levels were unavailable, the HScore could not be accurately calculated in five cases; therefore, analyses were conducted in 42 patients. All seven cytokines, including M-CSF (r = 0.80), total IL-18 (r = 0.78), LIF (r = 0.76), basic FGF (r = 0.72), CXCL9 (r = 0.68), IL-12 (p40) (r = 0.64), and active IL-18 (r = 0.58), showed significant positive correlations with the HScore (all p < 0.0001) (**Table 3**).

**Table 3:** The Correlations between serum cytokines associated with active IL-18 and HScore in AOSD patients (after Bonferroni`s correction).

Cytokines	Correlation coefficient (95%CI)	p value
M-CSF	0.80 (0.65-0.89)	<0.0001^*^
Total IL-18	0.78 (0.61-0.88)	<0.0001^*^
LIF	0.76 (0.58-0.87)	<0.0001^*^
Basic FGF	0.72 (0.53-0.84)	<0.0001^*^
CXCL9	0.68 (0.47-0.82)	<0.0001^*^
IL-12(p40)	0.64 (0.41-0.80)	<0.0001^*^
Active IL-18	0.58 (0.33-0.76)	<0.0001^*^

Basic FGF, basic fibroblast growth factor; CI, confidence interval; CXCL, chemokine (C-X-C motif) ligand; IL, interleukin; LIF, leukemia inhibitory factor; M-CSF, macrophage colony-stimulating factor.

* means there is a significant difference at p<0.0007.

### 3.6 Evaluation of the diagnostic biomarkers for AOSD

**Figure 5:**
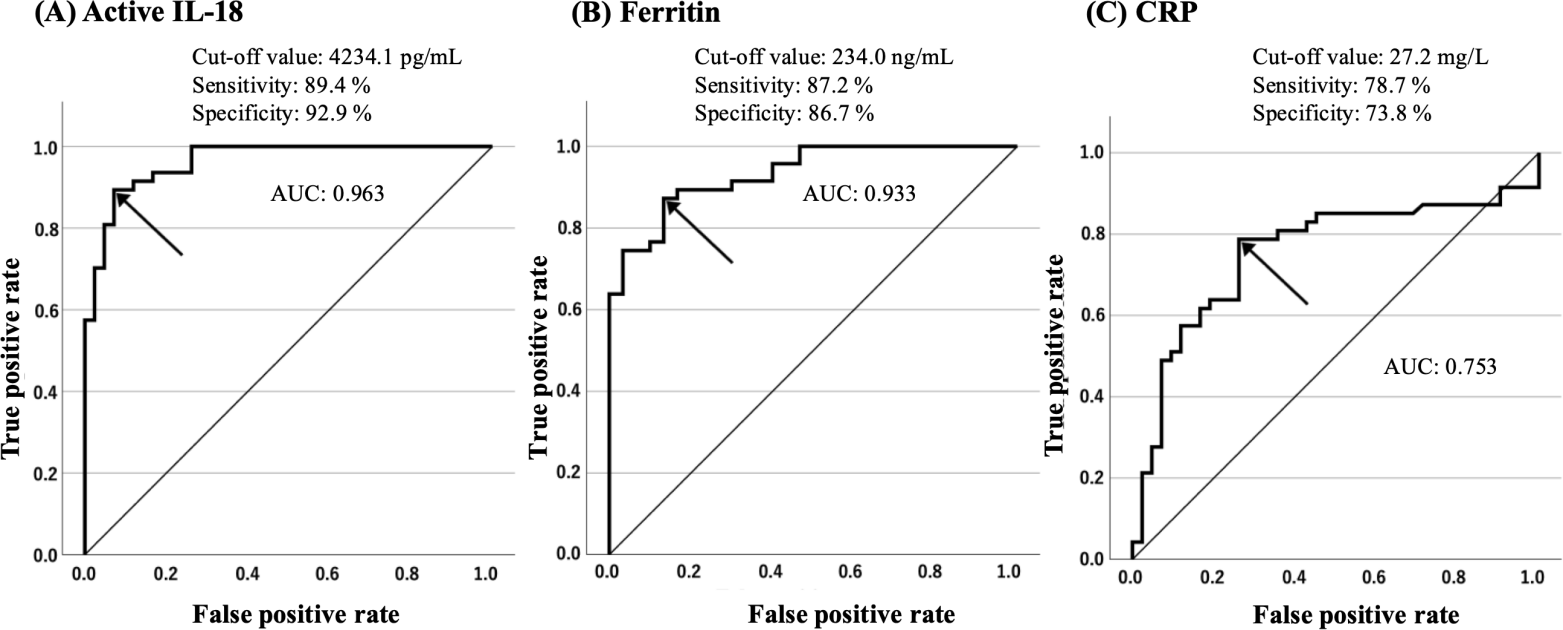
ROC curves illustrating the cutoff values and diagnostic discrimination of active IL-18, FT, and CRP between AOSD and RA. ROC curves showing the cutoff values, sensitivity, specificity, and overall diagnostic performance of **(A)** active IL-18, **(B)** FT, and **(C)** CRP in distinguishing AOSD from RA. Among the three clinical markers, active IL-18 was the most accurate for distinguishing AOSD from RA. AOSD, adult-onset Still’s disease; AUC, area under the curve; IL, interleukin; RA, rheumatoid arthritis; ROC, receiver operating characteristic.

**Figure 5** illustrates the results of logistic regression analysis distinguishing AOSD from RA, including the sensitivity, specificity, and AUC. Among the biomarkers evaluated, active IL-18 (> 4,231.1 pg/mL) demonstrated the highest diagnostic performance for distinguishing AOSD from RA, with a sensitivity of 89.4%, specificity of 92.9%, and an AUC of 0.963, outperforming ferritin and CRP.

## 4 Discussion

There is growing evidence for the involvement of inflammasomes in the pathophysiology of AOSD (8, 19, 20). Inflammasome activation comprises a two-step process involving the priming step, followed by the activation step, resulting in the caspase-1 activation, leading to the cleavage and release of active forms of IL-1ß and IL-18, which are associated with AOSD-related autoinflammatory conditions (21). These findings suggest that the serum level of active IL-1ß or IL18 could be a sensitive biomarker for inflammasome activation status in AOSD.

IL-18 is involved in inflammasome activation and macrophage- and natural killer cell-mediated innate immune responses, and it has been recognized as a key cytokine in diseases characterized by cytokine storms, such as AOSD and systemic juvenile idiopathic arthritis (22). However, most circulating IL-18 is neutralized by its binding protein, IL-18BP (23, 24), and the measured “total IL-18” represents both the bound and active forms. Among these, active IL-18 is believed to possess biological activity (9). According to Girard et al., total IL-18 is elevated not only in AOSD but also in other systemic autoinflammatory disorders, including familial Mediterranean fever, whereas free IL-18 appears to be selectively elevated in AOSD, suggesting its potential value as a disease-specific biomarker (25). Furthermore, Girard et al. reported that free IL-18 was detected specifically in patients with AOSD, in contrast to other rheumatic diseases such as RA, psoriatic arthritis, ankylosing spondylitis, and systemic lupus erythematosus (26). This study demonstrated that serum levels of active IL-18 in patients with AOSD were significantly elevated compared with those in patients with RA, patients with FMF, and HCs. Moreover, the serum levels of active IL-18 were significantly correlated with disease activation markers, such as Pouchot score or ferritin level. These findings suggest that elevated levels of active IL-18 may reflect the inflammasome activation status in AOSD. Moreover, ROC curve analyses comparing serum ferritin and CRP levels in patients with AOSD showed that active IL-18 was a significantly more sensitive and specific marker for diagnosing AOSD.

In our study, serum active IL-18 levels significantly decreased after treatment for AOSD. However, elevated levels were observed in a patient with underlying hepatitis C virus reactivation despite the absence of active AOSD. Proinflammatory cytokines, including IL-18, have been shown to play an important role in the control of viral infections (27). Chattergoon et al. (28) reported that serum IL-18 levels increase during the early phase of detectable hepatitis C virus (HCV) RNA, return to baseline upon viral clearance in cases of spontaneous resolution, and remain persistently elevated in cases of chronic infection. Sharma et al. (29) further demonstrated that serum IL-18 levels are elevated in patients with chronic hepatitis C and show a strong positive correlation with hepatic inflammatory activity and necrotic changes in liver tissue. Although the elevated active IL-18 level observed in one treated patient with AOSD in the present study contrasts with the overall trend, it may reflect physiological changes associated with hepatitis C virus reactivation.

IL-18 acts synergistically with IL-12 to stimulate Th1 responses through the induction of IFN-γ production (30). We previously reported that IL-12 (p40) may be involved in the inflammatory pathogenesis of AOSD (10). In our study, serum levels of active IL-18 were significantly higher in patients with AOSD than in controls and were correlated with IL-12 (p40), M-CSF, basic FGF, LIF, and CXCL9, but not with IFN-γ levels. Conflicting data regarding IFN-γ levels in AOSD have been reported, with some studies demonstrating the elevated concentrations of IFN-γ (31–33), whereas others reported lower IFN-γ levels in AOSD than in RA (34). More recently, higher levels of CXCL9, an IFN-γ-induced chemokine, have been reported as a biomarker for stratifying patients with Still’s disease-associated MAS (31, 35, 36). Our data showed that serum levels of active IL-18 correlated with those of CXCL9 and HScore in patients with AOSD. High concentrations of M-CSF, a growth factor critical for macrophage differentiation and activation, are observed in the plasma of patients with AOSD (37, 38). Recent genome-wide association studies have suggested that genetic variants near the CSF1 gene, which encodes M-CSF, are associated with AOSD and are linked to elevated M-CSF levels and systemic disease outcomes (39). Basic FGF, also known as FGF2, is induced by IL-1β in human osteoblasts and fibroblasts (40) and is a potent angiogenic factor that is released after tissue injury and during inflammation (41). Koga et al. (42) reported that basic FGF may serve as a potential biomarker for distinguishing adult-onset Still’s disease from sepsis. LIF is a pleiotropic cytokine belonging to the IL-6 superfamily (43). Circulating LIF levels are elevated in systemic inflammatory conditions such as septic shock (44) and are also increased in the synovial fluid of patients with rheumatoid arthritis, where they correlate with IL-1β, IL-6, and IL-8 (45). LIF is produced by monocytes/macrophages, synovial fibroblasts, endothelial cells, and chondrocytes (45), and cultured human synovial cells are known to produce LIF in response to stimulation with IL-1 or TNF-α (46). Furthermore, Villiger et al. reported that LIF induces the gene expression of IL-1β and IL-6 in human rheumatoid arthritis synovial cells (47). Although LIF may exacerbate inflammatory responses, its role in adult-onset Still’s disease remains unclear. Further case evaluation is needed to determine the relationship between serum levels of active IL-18 and macrophage status for the management of patients with AOSD and MAS.

In addition, recent data suggest that IL-18 is involved in the pathogenesis of MAS (48, 49). Interestingly, IL-18 levels are highly elevated in patients with NLRC4 cryopyrinopathy, in whom MAS and splenomegaly are frequently observed, and inflammasome activation or active IL-18 appear to be involved in these clinical manifestations (50, 51). Although the levels of active IL-18 were significantly elevated in patients with AOSD with splenomegaly, there was no significant difference between patients with AOSD with and without MAS. The number of patients with AOSD was limited, and a larger number of cases is needed to elucidate the relationship between the circulating active form of IL-18 and these organopathies in AOSD.

This study has several limitations. First, it was a cross-sectional study and, therefore, subject to the inherent limitations of the study design. Larger prospective studies are required to elucidate the association between serum active IL-18 levels and AOSD progression and prognosis. Second, the study population comprised exclusively Japanese individuals. Therefore, it remains unclear whether these findings can be generalized to other ethnic groups. Third, multiple serum cytokines were measured in patients with AOSD but not in the control groups, including patients with RA, FMF, or HCs. Consequently, we were unable to assess the discriminative performance of inflammatory cytokines using ROC analysis. In addition, baseline characteristics such as age and sex differed among the study groups. Furthermore, although the RA patients and FMF patients in the control group had clinically active disease, a substantial proportion were treated with disease-modifying antirheumatic drugs and/or biologic agents, which may have influenced cytokine levels. Fourth, the sample size was small, and the biomarkers were measured at only two time points. Although post-treatment samples from patients with AOSD were collected during inactive disease, the timing was not clearly defined. Moreover, the treatment regimens were not standardized, and serum active IL-18 levels may have been affected by the medications used. Further studies with larger cohorts and analyses of innate immune cell populations are warranted to elucidate the mechanisms underlying elevated levels of serum active IL-18.

In conclusion, the present study demonstrated that serum levels of the cleaved form of IL-18 were significantly elevated and correlated with disease activity, such as Pouchot score and serum levels of ferritin, in patients with AOSD. We also demonstrated that cleaved form of active IL-18 levels were associated with clinical manifestations such as skin rash and splenomegaly and correlated with cytokines implicated in AOSD, including IL-12(p40), M-CSF, basic FGF, LIF, and CXCL9. These findings suggest that specific clinical features and serum cytokine profiles may help identify a subset of patients with elevated circulating active IL-18 and enhanced inflammasome activity. This may contribute to the development of personalized treatment strategies for AOSD and further supports the utility of active IL-18 as a biomarker of disease activity and inflammasome activation.

## Data Availability

The original contributions presented in the study are included in the article/**Supplementary Material**. Further inquiries can be directed to the corresponding author.
