# Supplementary material for: Association of elevated serum active IL-18 levels with cytokine profiles and clinical features in adult-onset Still’s disease
Source: Front Immunol. 2026 Apr 28;17:1759584. doi: 10.3389/fimmu.2026.1759584 (PMC13160908; doi:10.3389/fimmu.2026.1759584)
Supplement: Supplementary file 1 [file Table1.docx]

**Supplementary Table 1**

**Demographic and clinical characteristics of AOSD patients at blood sampling after treatment.**

| **Variables**  **AOSD patients after treatment** | | **n=18** |
| --- | --- | --- |
| Female, n (%) | 12 (66.7) | |
| Age (years), median (IQR) | 52.5 (35-73) | |
| Interval between initial blood sampling and follow-up sampling (month), median (IQR) | 27 (19.8-57) | |
| Ferritin (ng/mL), median (IQR) | 37.5 (22-111.8) | |
| CRP (mg/L), median (IQR) | 0.4 (0.1-1.0) | |
| WBC (/μL), median (IQR) | 6,800 (6,100-8,775) | |
| AST (U/L), median (IQR) | 21 (16.3-30.8) | |
| ALT (U/L), median (IQR) | 22 (13.5-49) | |
| Use of PSL, n (%) | 17 (94.4) | |
| PSL dose (mg/day), median (IQR) | 10 (5.0-17.5) | |
| Concomitant use of immunosuppressant, n (%) | 10 (55.6) | |
| MTX, n (%) | 5 (27.8) | |
| CyA, n (%) | 7 (38.9) | |
| TAC, n (%) | 1 (5.6) | |
| Concomitant use of biological agent, n (%) | 0 (0.0) | |

All data are expressed as median (IQR), or numbers (percentages).

AOSD: adult-onset Still's disease, ALT: alanine aminotransferase, AST: aspartate aminotransferase, CRP: C-reactive protein, CyA: cyclosporine A, IQR: interquartile range, MTX: methotrexate, PSL: prednisolone, RA: rheumatoid arthritis, TAC: tacrolimus, WBC: white blood cell.
