# Supplementary material for: Association of elevated serum active IL-18 levels with cytokine profiles and clinical features in adult-onset Still’s disease
Source: Front Immunol. 2026 Apr 28;17:1759584. doi: 10.3389/fimmu.2026.1759584 (PMC13160908; doi:10.3389/fimmu.2026.1759584)
Supplement: Supplementary file 2 [file Table2.docx]

**Supplementary Table 2**

**Demographic and Clinical Characteristics of Patients with RA and HCs at the Time of Blood Sampling.**

| **Variables**  **RA patients** | **n=42** | | **p-value** |
| --- | --- | --- | --- |
| Female, n (%) | | 31 (73.8) | 0.64 |
| Age (years), median (IQR) | | 66 (58.3-70) | <0.001^*^ |
| Use of MTX, n (%) | | 21 (50.0) |  |
| Concomitant use of PSL, n (%) | | 18 (42.9) |  |
| Concomitant use of other csDMARDs, n (%) | | 6 (14.3) |  |
| Concomitant use of bDMARDs, n (%) | | 15 (35.7) |  |
| CRP (mg/L), median (IQR) | | 5.5 (3.5-30.7) |  |
| DAS28-CRP, median (IQR) | | 2.8 (2.1-4.00) |  |
| FMF patients | | **n=9** |  |
| Female, n (%) | | 4 (44.4) | 0.26 |
| Age (years), median (IQR) | | 43 (23-52) | 0.53 |
| Typical FMF, n (%) | | 1 (11.1) |  |
| Use of colchicine, n (%) | | 4 (44.4) |  |
| Use of canakinumab, n (%) | | 1 (11.1) |  |
| CRP during an attack (mg/L), median (IQR) | | 18.7 (8.3-51.8) |  |
| SAA during an attack (μg/dL), median (IQR) | | 46.2 (8.2-219.5) |  |
| **HCs** | | **n=26** |  |
| Female, n (%) | | 16 (61.5) | 0.61 |
| Age (years), median (IQR) | | 32.5 (26.3-44.5) | 0.04^*^ |

All data are expressed as median (IQR), or numbers (percentages). * means there is a significant difference at p<0.05. The p-values represent the results of comparisons between each group and the AOSD group for the corresponding variables.

bDMARDs: biologic disease-modifying antirheumatic drugs, CRP: C-reactive protein, csDMARDs: conventional synthetic disease-modifying antirheumatic drugs, DAS28: Disease Activity Score-28, FMF: familial Mediterranean fever, HC: healthy control, IQR: interquartile range, MTX: methotrexate, PSL: prednisolone, RA: rheumatoid arthritis, SAA: serum amyloid A, WBC: white blood cell.
