# Supplementary material for: Association of elevated serum active IL-18 levels with cytokine profiles and clinical features in adult-onset Still’s disease
Source: Front Immunol. 2026 Apr 28;17:1759584. doi: 10.3389/fimmu.2026.1759584 (PMC13160908; doi:10.3389/fimmu.2026.1759584)
Supplement: Supplementary file 3 [file Table3.docx]

**Supplementary Table 3**

**Serum concentrations of active IL-18 and 6 correlated cytokines**

| **Cytokines** | **Serum concentration** |
| --- | --- |
| M-CSF (pg/mL) | 184.2 (135.9-208.7) |
| Total IL-18 (pg/mL) | 10,307.4 (7,355.0-12,628.1) |
| LIF (pg/mL) | 154.1 (119.4-186.6) |
| Basic FGF (pg/mL) | 76.7 (64.8-85.0) |
| CXCL9 (pg/mL) | 241.4 (89.3-804.0) |
| IL-12(p40) (pg/mL) | 254.2 (215.3-302.3) |
| Active IL-18 (pg/mL) | 8,592.7 (6,690.3-10,048.6) |

Basic FGF; basic fibroblast growth factor, CXCL; chemokine (C-X-C motif) ligand, IL; interleukin, LIF; leukemia inhibitory factor, M-CSF; macrophage colony-stimulating factor.
